# Challenges and strategies for maintaining nutrition services in the Democratic Republic of Congo during COVID-19: a qualitative study

**DOI:** 10.3389/frhs.2025.1551131

**Published:** 2025-06-24

**Authors:** Marc Bosonkie, Benito Kazenza, Rawlance Ndejjo, Marie-Claire Muyer, Eric Mafuta, Ruphin Mbuyi, Branly Mbunga, Paul-Samsom Lusamba, Olufunmilayo I. Fawole, Mala Ali Mapatano

**Affiliations:** ^1^ Department of Nutrition, Kinshasa School of Public Health, School of Medicine, University of Kinshasa, Kinshasa, Democratic Republic of Congo; ^2^ Department of Disease Control and Environmental Health, School of Public Health, College of Health Sciences, Makerere University, Kampala, Uganda; ^3^ Department of Health Management and Policy, Kinshasa School of Public Health, School of Medicine, University of Kinshasa, Kinshasa, Democratic Republic of Congo; ^4^ Social Protection, Centre National d'Appui au Développement et à la Participation Populaire, Civil Society Organizations, Kinshasa, Democratic Republic of Congo; ^5^ Department of Biostatistics and Epidemiology, Kinshasa School of Public Health, School of Medicine, University of Kinshasa, Kinshasa, Democratic Republic of Congo; ^6^ Department of Epidemiology and Medical Statistics, Faculty of Public Health, College of Medicine, University of Ibadan, Ibadan, Nigeria

**Keywords:** COVID-19, challenges, solutions, maintaining nutrition services, DRC

## Abstract

**Background:**

The rapid spread of COVID-19 forced governments to urgently implement non-pharmaceutical measures to stop the surge. These restrictions disrupted the provision of nutrition services. This study identified challenges faced by nutrition services using the six components of the health system and preventive strategies that can strengthen nutrition interventions during future outbreaks.

**Methods:**

A multiple-case qualitative study was carried out. Purposive sampling was used for recruitment of participants. 57 key informants were selected based on their role in the Nutrition sector at different levels of the health pyramid. The interview guide incorporated nutrition leadership, financing, workforce, infrastructure and commodities, service delivery and information system. Each topic had subtopics on challenges and adaptations. All transcripts were exported to Atlas Ti v22, and thematic analysis was conducted.

**Results:**

Initially excluded from the national COVID-19 response, nutrition services were later integrated through advocacy by the National Nutrition Program. Despite limited funding, the government maintained support, and health workers adapted with flexible staffing approaches. Commodity shortages, including Ready-to-Use Therapeutic Food, led to the use of locally produced substitutes. Movement restrictions and fear of infection disrupted essential services such as growth monitoring and immunization. To sustain access, mitigation strategies were implemented, including tailored education, modified weighing methods, and decentralized care. Key innovations included rapid registration with anthropometric protocols, additional service points for child health activities, double-weighing scales to reduce contact, crowd control during Growth Monitoring Promotion, community-based service delivery, and improved digital integration.

**Conclusions:**

COVID-19 disrupted all pillars of nutrition services in the DRC but also spurred innovation. Institutionalizing adaptive strategies, securing sustainable funding, and supporting local Ready-to-Use Therapeutic Food production are essential to strengthen resilience and ensure continuity of nutrition services in future health emergencies.

## 1 Introduction

On 11 March 2020, the World Health Organization (WHO) declared COVID-19 to be a pandemic (1). As of 3 October 2023, the pandemic affected more than 66 million people worldwide and caused more than six million deaths^1^. Its rapid spread forced governments and organizations to take drastic non-pharmaceutical measures deemed promising to curb the surge, such as lockdowns, travel restrictions, school closures, mass gathering restriction, etc. (2). These restriction measures created a disruption of essential non-COVID services that went beyond causing direct mortality and morbidity (3).

Essential non-COVID services for all areas included reproductive, maternal, newborn, child and adolescent health, prevention and management of communicable diseases, treatment for chronic diseases to avoid complications, and addressing emergencies (4). Studies on these non-COVID-related services focused on maternal and perinatal health, immunization, tuberculosis and chronic non-communicable diseases (5–9). Conversely, services such as adolescent health, health of the elderly (older than 60 years) and nutrition received little attention, despite being foundational to primary health care and vital for protecting population health (10). Globally, existing studies on nutrition services (NS) either focused on strategies to ensure continuity of nutritional management with COVID-19 cases (11, 12) or on the disruptions and restorations of, and adaptations to health and nutrition services' delivery (12).

Yet, the pandemic also disrupted nutrition services delivery across Africa. A study conducted in East and Southern Africa highlighted that Vitamin A supplementation was severely affected, as less than half the number of children received the recommended dose. In response to the disruption of childcare delivery, 21 countries in the region initiated the use of family mid-upper arm circumference measurements (13). An analysis projected that Sub-Saharan Africa will in the long run experience further increases in child stunting and wastingdue to the pandemic (14).

In the Democratic Republic of Congo (DRC), Nutrition-related services in children include growth monitoring promotion^2^, infant and young child feeding^3^, community-based nutrition^4^ and integrated management of acute malnutrition^5^. Growth monitoring promotion^6^ is a package of preventive and promotional care offered to children from birth to five years of age, with a view to ensuring their steady growth and development. Growth monitoring promotion involves anthropometric measurements, collective health and nutrition education, screening for acute malnutrition and for other killer diseases, guidance, cooking demonstration, vitamin A supplementation and immunization^7^. Community-based nutrition^8^ places particular emphasis on community organization, community diagnosis, capacity for local plans to combat malnutrition and mobilize local resources. These growth monitoring promotion and community-based nutrition make infant and young child feeding an essential activity for the National Nutrition Program^9^. In addition, integrated management of acute malnutrition integrates malnutrition into the national health system with responsibilities shared between the health facilities and communities^10^.

Despite the critical role of preventive, promotional, and nutrition management activities in primary health care, limited research has examined the impact of COVID-19 on these essential services. A comprehensive approach is necessary to understand and address the disruptions caused by the pandemic. To achieve this, our study applies the WHO health system framework, which consists of six key components: (1) leadership and governance, (2) financing, (3) workforce, (4) infrastructure and commodities, (5) nutrition service delivery, and (6) nutrition information systems (15).

The health system of the DRC, as well other countries of sub-Saharan Africa, will likely continue to be confronted by restless emerging global and local emergencies as with the 15 episodes of Ebola outbreaks (16), approximately three decades of armed conflicts in eastern DRC (17) and the potential for emerging and reemerging infectious diseases and climate change (18). The possibility of future outbreaks cannot be ruled out. Also, with fragile health systems, most African countries are likely to be overwhelmed in event of another pandemic (19). Hence, it is crucial to analyze disruption and document the lessons learned to ensure the maintenance of nutrition services during future crisis or events.

Given that the number of confirmed COVID-19 cases in the DRC during the first four waves (from March 2020 to January 2022) was highest in Haut-Katanga, Kinshasa, Kongo-central and North Kivu (20). Therefore, our study focused on these high-disease-burdened provinces. This paper aimed to identify challenges faced by nutrition services across the six building blocks of the health system and preventive strategies undertaken to strengthen nutrition interventions during future outbreaks.

## 2 Materials and methods

### 2.1 Study area

The study was conducted in four provinces of the DRC: Haut-Katanga, Kinshasa, Kongo-central and North Kivu. They represent the four provinces most affected by COVID-19 in the DRC from March 2020 to January 2022 (20).

### 2.2 Study design

A multiple-case qualitative study was carried out at different levels of the DRC health system. Key informants (KI) were interviewed.

### 2.3 Participants, sampling and recruitment

**Figure 1:**
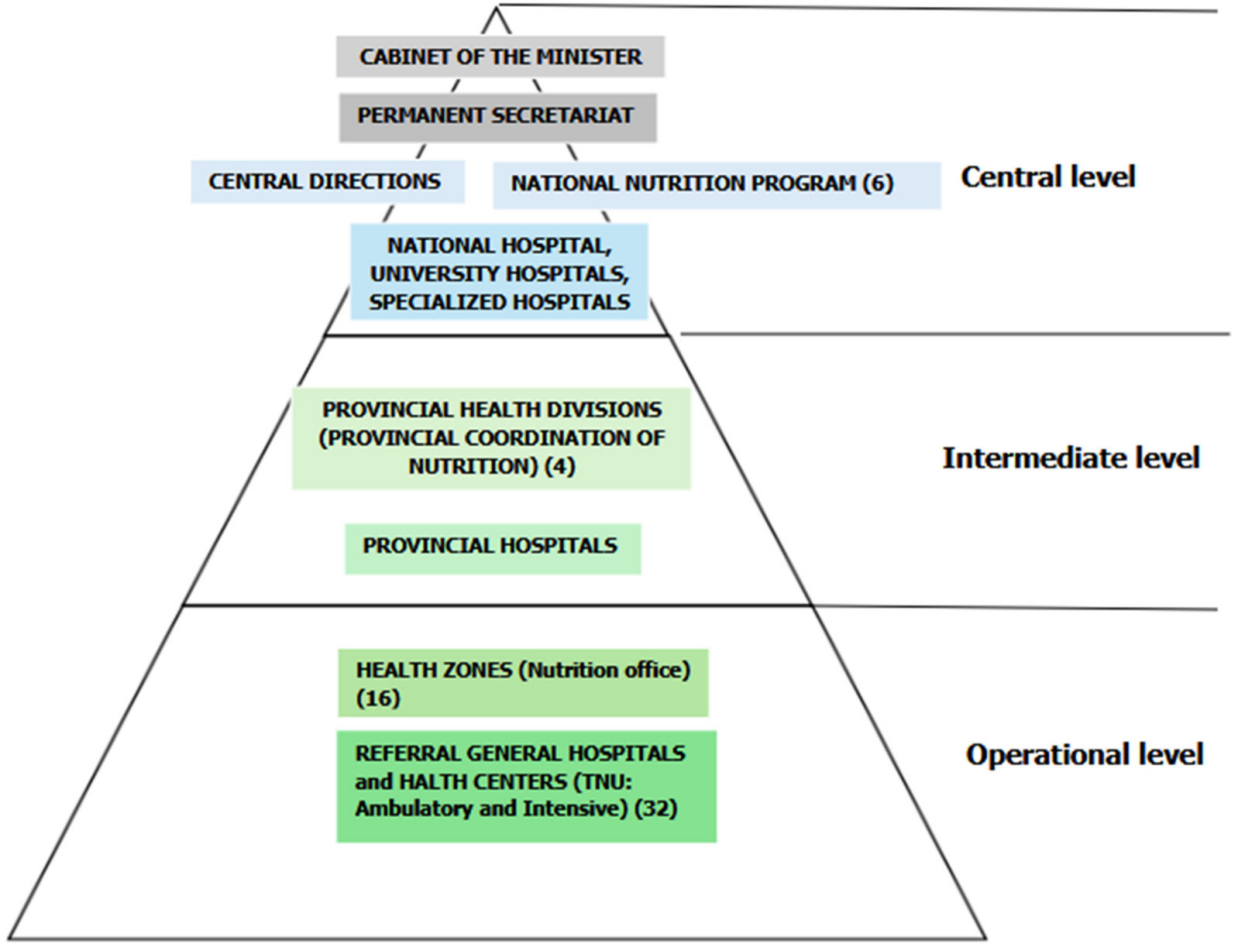
DRC health pyramid. Figure 1 describes the DRC health pyramid and number of participants per level.

We included participants from both government and non-government sectors. From government sectors, each level of the health system (national, provincial, and operational) was represented (21). Purposive sampling was used for the recruitment of participants based on their roles in the Nutrition sector at the three levels of the health system. At the national level, the National Program of Nutrition (PRONANUT) represents one of the sectoral programs and operates under the Permanent Secretariat. PRONANUT works closely with the provincial level through the Provincial Coordination of Nutrition. The provincial level in the DRC serves as the support level for the operational level. In this regard, we selected a non-governmental representation (Nord-Kivu and Kongo-Central) and a governmental representation (Kinshasa and Haut-Katanga) meaning four at the provincial level. The operational level includes health zones and therapeutic nutritional units (TNU), which are based in hospitals or at health centers (22). We recruited six participants at the national level (governmental and non-governmental sectors) and four at the provincial level (governmental or non-governmental sectors) (see Figure 1). At the operational level, we selected four health zones in each province (two rural and two urban) with one participant from each, meaning 16 participants were interviewed. For the TNUs, we included one participant for health facility activities and one for community activities, thus, 32 participants for all TNUs. A total of 57 KI were interviewed as one participant declined to be interviewed. KI were Managers at the central level, officials from the Provincial Health Division or the Provincial Coordination of the Nutrition Program, as well as community health workers and nutritionists at the health zone level or nutrition officers and providers in the nutrition units of hospitals or health centers (see Figure 1).

### 2.4 Data collection

The interviewers were public health specialists or physicians with experience in qualitative data collection. We used a semi-structured interview guide to facilitate the discussion and mobile phones to audio record the interviews. The interview guide was written in French and translated into local languages (Lingala, Kikongo and Swahili). The interview guide incorporated several topics comprising leadership and governance, financing, health workforce, infrastructure and commodities, health service delivery, and health information system. Each topic had subtopics on challenges and adaptations. Before data collection began, a three-day training session was organized for interviewers on qualitative research interviews and the tool to be used. The quality of the interviews was ensured by training the thirteen interviewers (three per province plus the principal investigator) in interview techniques, pre-testing the interview guides and discussions with the local communities. Four provincial supervisors were selected and trained for three days in Kinshasa. Subsequently, a two-day pretest was carried out in Kinshasa. Then, each provincial supervisor was responsible for recruiting two local interviewers in their assigned province. The fourth day in the province was dedicated to pretesting at two levels (provincial and operational). The fifth day of training was devoted to a debriefing session in the respective provinces. Data were collected during February 2022.

Face-to-face interviews were conducted with strict observance of COVID-19 standard prevention operating procedures such as physical distancing and wearing masks. Interviewers visited the offices of the informants at a time convenient to them to conduct the KI interviews, which lasted between 45 and 80 min. A summary of the interview was documented by the interviewers after each interview. The interviewers themselves transcribed the audio recordings, respecting the verbatim and local languages. Two senior qualitative researchers reviewed the transcriptions and coding to ensure harmonization and consensus. The saturation was assessed through the daily feedback combined with parallel data analysis. Data processing targets checking the redundancy of responses among respondents and across successive interviews until no new themes and information emerge (23).

### 2.5 Data analysis

To identify key themes emerging from the interviews, a deductive-thematic analysis was used and based on the theoretical framework proposed by Kruk et al. Indeed, this approach was preferred to better focus on the experiences and perspectives of our KIs. Then, all transcripts were exported to Atlas Ti version 22. All data collected and analyzed were all conducted according to WHO health system framework. and analysis used the six steps of thematic analysis described by Braun and Clarke (24), which are: (1) data familiarization: all the transcripts were read for an overall understanding of the data, (2) generating initial codes: codes addressing research questions were identified, (3) identifying preliminary themes: the initial codes were synthesized into wider themes, (4) reviewing themes: the themes from the frontline health workers and policy-makers were reviewed and synthesized, (5) defining and naming themes: we drew up figures for thematic analysis where data for each theme were collected from all interview transcripts, and (6) reporting. Reports were developed with quotes from the interviews and themes identified. Consolidated criteria for reporting qualitative research guidelines led the design and reporting of our study (25).

### 2.6 Ethical considerations

The study was approved by the Kinshasa School of Public Health Ethics Committee with the number ESP/CE/198/2020. The studies were conducted in accordance with the local legislation and institutional requirements. The participants provided their written informed consent to participate in this study. Written informed consent was obtained from the individual(s) for the publication of any potentially identifiable images or data included in this article.

## 3 Results

Table 1 presents the characteristics of the participants of the 57 KI interviews conducted. More than a third of participants were aged between 41 and 50. More than three quarters were male. Overall, almost 9 out of 10 had at least five years' experience in nutrition services. Apart from one participant at national level, there was equal representation across the levels.

**Table 1:** Characteristics of participants.

Characteristics	*n*
Age (years)	
30–40	12
41–50	20
51–60	17
60+	8
Sex	
Male	45
Female	12
Year of work experience	
<5	6
≥5	51
Level of the health system	
National level	6
Provincial level	4
Operational level	47

A total of 57 participants were included in the study. The majority were aged between 41 and 60 years. Most participants were male (*n* = 45; 78.9%), and the vast majority (*n* = 51; 89.5%) reported having five or more years of professional experience in the health sector. Regarding their level of engagement within the health system, 47 participants (82.5%) operated at the operational level, while 6 (10.5%) and 4 (7.0%) worked at the national and provincial levels, respectively.

The results section is described following the six components of the health system comprising nutrition leadership and governance, nutrition financing, nutrition workforce, nutrition infrastructure and commodities, nutrition services delivery and nutrition information system. For each topic, challenges and adaptations are presented.

### 3.1 Theme 1: leadership and governance

The COVID-19 response was organised into pillars, with each pillar reporting to a section. However, a PRONANUT officer reported that the first COVID-19 response plan, written on 8 March 2020, did not include nutrition as a pillar, not even as a component of one of the pillars of the response. He said:

“I don't understand how nutrition was overlooked in the COVID-19 response. Before treatment, it's essential to assess nutritional status. Malnourished patients can't receive standard doses and require adjustments.” (KI-1, PRONANUT).

A manager in nutrition mentioned that as nutrition was not included in the response at the start of the pandemic, the continuity of services was not yet the concern of the national PRONANUT. At the time, the national PRONANUT focused on advocacy in order to be recognised as a pillar in the response (acting as a sub-committee of the medical care committee). This recognition in mid-April 2020 was an activator for PRONANUT, restructuring the COVID-19 nutrition response and the continuity of nutrition services at different levels (national, provincial and operational).

“…Being included as a pillar in the medical care commission marked the beginning of PRONANUT's involvement across all levels. It was a wake-up call that pushed us to engage in the COVID-19 response while ensuring routine activities followed essential health service guidelines…” (KI-2, PRONANUT).

### 3.2 Theme 2: financing

The financing of the national program of nutrition in the DRC involves multiple sources and strategies with a very low level of public funding. Informants (4/4) indicated that, during COVID-19, there was no specific fund for the maintenance of nutrition services. A nurse at the TNU said,

“We had no budget to maintain routine services. Funding was focused on nutrition care for COVID-19 patients. During the first wave, routine activities lacked support, with shortages of PPE and RUTF across many health zones” (KI-2, PRONANUT Nord-Kivu)

However, respondents (3/4) recognized that the government reallocated the domestic health budget to ensure the financing. For example, the government continued to provide some financial resources to health facilities and pay health workers throughout the pandemic as noted below:

“The government's contribution, as budgeted, mainly covered health worker salaries and supplies. Public sector staff and Ministry agents were paid regularly, which significantly helped sustain essential health services”. (KI 8, PRONANUT Haut-Katanga).

### 3.3 Theme 3: service workforce

• Several participants (3/4) highlighted that during the COVID 19 pandemic, the workload remained the same or even greater in different departments, with a reduced number of staff at different levels of the health pyramid. This led to an overload of work at the national, provincial and operational levels. One informant said.

“Due to the Gombe lockdown, our national office moved to a small room at Kintambo hospital, allowing only the Director and division heads to be physically present. At the operational level, a single provider in a TNU had to handle growth monitoring and care for all malnourished children—an overwhelming workload”. (KI 7, National level).

A staff member stated that the strategy was applied at the normative level: rotating staff, with some online and others face-to-face. For the operational level, crisis schedules were drawn up at various levels of PRONANUT to manage social distancing and the number of staff per working day in order to reduce the workload. He said,

“Increasing the number of staff in TNUs to two or three, depending on the case, was a bit of a relief. Tasks were divided into two so we could take a breather” (KI 11, PRONANUT, Kongo central).

• A community health worker described that, for community activities, community health workers had been involved in community screening of cases of malnutrition, as well as for nutritional assessment before starting COVID-19 treatment at home (for those cases able to be treated at home).
• “*During the COVID-19 pandemic, we went into households with our mid-upper-arm circumference (MUAC) tapes to assess the nutritional status of patients due to start COVID-19 treatment. The nurses in the health centers did not come to do this work”.* (KI 25, CHW)

### 3.4 Theme 4: infrastructure and commodities

Among other things, KIs (4/4) from TNUs mentioned that challenges are reflected in the lack of didactic tools to support nutrition education, sometimes the lack of suitable registers and the failure to renew measuring tools (scales, etc.), even lack of underpants to weigh the children during growth monitoring promotion. In fact, to protect the children, everyone had to find their own pair of underpants to use for weighing. Unfortunately, mothers often did not bring appropriate pants, so the children could not be weighed. One respondent said,

“The children were not weighed during the COVID-19 period, even though our children's growth is to be monitored by weighing them. Which was a huge gap”. (KI 12, PRONANUT, Haut-Katanga).

In terms of curative treatment for malnutrition, the challenges were even greater. The lack of RUTF for some health care facilities, untimely shortages of RUTF for others, the lack of complementary inputs (medicines), the low economic level of households attending care units, and the lack of attention paid by health care facilities to the nutrition services in relation to the costs associated with running the service, are among the challenges mentioned by respondents (3/4).

One respondent said:

“We face many challenges in caring for malnourished children. Resources are often limited—we sometimes manage moderate cases, but often can only provide nutritional education and guidance to mothers, as we lack the means for full treatment”. (KI 30, NTU, Nord-Kivu).

A health zone officer highlighted the support of the maintenance of services, especially for nutrition. The Ministry of Health, with the technical and financial support of the WHO and UNICEF, launched a major campaign in favour of the detection of cases in the community. This was to save certain community activities that could no longer take place at the hospital due to a lack of rooms. She said,

“With the support of UNICEF and other partners, we were able to continue community activities. In Nsele, for example, CBN continued, thanks to the support of our partners. We’ve even certified some health areas as CBN villages”. (KI 36, Health zone, Kinshasa).

The findings from the KIs highlighted the importance of promoting local RUTF.

“…Even though it was not standardized, we found that many TNUs had developed one or more local foods as therapeutic alternatives during the COVID-19 period, using their own efforts. Some had begun this process before COVID-19, but the advent of COVID-19 meant that some TNUs that were not [otherwise] supported continued to treat our children with local foods….” (KI 26, TNU, Kongo-central).

There was also implementation of regular cooking demonstrations for mothers of malnourished and normal children in the community and during the growth monitoring promotion.

“…We have helped many mothers to master the combinations of foods to prevent malnutrition in normal children but also to use the combination of certain local foods that we have found to be effective in treating their malnourished children…” (KI 27, Haut-Katanga).

### 3.5 Theme 5: service delivery

According to respondents, during the COVID-19 period, the activities were significantly affected. Non-pharmaceutical measures introduced to limit the spread of the COVID-19, including limiting gatherings of more than 20 people and social distancing, affected the number of mothers attending the growth monitoring promotion. According to respondents, this was due, in part, to the instruction in the form of a directive received from their respective health zones, not to exceed 20 mothers and children during growth monitoring promotion *sessions*; on the other hand, the health facilities had to fall into line and apply the measures dictated by the country. In addition, the respondents also mentioned that some mothers refused to go to the facility for fear of contracting the disease and/or exposing their child to the same risk.

Some respondents (2/4) said,

“We had to limit the number of children to be vaccinated so that we could respect the social distancing measures and those linked to gatherings”. (KI 40, TNU, Kinshasa)

“During the COVID-19 pandemic, mothers were required to clothe their children with panties when weighing them”. (KI 41, NTU, Kongo-central)

The other negative effects of non-pharmaceutical measures on growth monitoring promotion included the inability of facilities to reach the expected target for certain antigens in the immunization schedule, following the measure restricting attendance of more than 20 people. Some children were not vaccinated during the period along with nutrition education no longer being adequately provided. The reason given by respondents was the need to quickly release mothers present at the growth monitoring promotion to avoid crowding. One respondent stated,

“In order to avoid exposing ourselves, the mothers and their children, we are starting to skip certain topics such as nutritional education during our growth monitoring promotions. We know it's important, but we have no choice”. (KI 42, NTU Kinshasa).

#### 3.5.1 Challenges with community-based nutrition during COVID-19

The COVID-19 pandemic had a negative impact on community-based nutrition activities in some settings with non-pharmaceutical measures such as limited contact, limited movement, confinement and social distancing. Here are statements from some respondents (2/4):

“It was difficult because we weren’t supposed to touch, we were supposed to be at a distance. As a result, community-based nutrition really suffered during that period of the pandemic. We weren’t supposed to go out in the field because it was difficult. Even in the vehicle, our boss said only one person in a vehicle with the driver, and anthropometric parameters you can’t do alone. So, it was really a pain”. (KI 44, Haut-Katanga).

Nevertheless, while community-based nutrition was successful in the context of COVID-19 in some settings, it was not the case in others due to the restriction of movement and fear of visits to households at the start of the pandemic. According to some respondents (3/4), these exacerbated cases of malnutrition.

“The detection of cases of malnutrition in the community, by community health workers, facilitated referral to the TNU. With COVID-19, there are many cases in the community, that's for sure. Families will come to TNU at a severe stage and generally with complications”. (KI 16, PRONANUT, Kinshasa).

#### 3.5.2 Challenges with the integrated management of acute malnutrition during COVID-19

During the COVID-19 pandemic, integrated management of acute malnutrition was adversely impacted in some TNUs. Indeed, while some facilities (2/4) had to report shortages of RUTF and other inputs required for integrated management of acute malnutrition, others mentioned the refusal of the mother and child to go to the care facility. These refusals were underpinned by stigmatization and the community's perception of her child's state of malnutrition. Households were afraid to use TNU because of prejudice.

“…there was an impact on inputs. That was really clear, because everything was blocked, nothing could enter the country and UNICEF had no inputs. This had a major impact on the management of malnourished cases”. (KI 46, TNU, Nord-Kivu).

“…I can say that many of them are at home, afraid that if they go to hospital, they’ll be told that this is a case of malnutrition caused by COVID-19, and yet that is false, very false. People are afraid to go to hospital, and that makes the disease worse”. (KI 47, TNU, Kongo-central).

#### 3.5.3 Common delivery interventions for maintaining nutrition services

To mitigate the effects of COVID-19 on nutrition activities in TNU, several strategies were put in place by way of guidance from PRONANUT, provincial nutrition offices, health zones and healthcare facilities. These strategies included prevention (nutrition education, screening) and treatment.

“The boss issued a note to limit the number of trainees and the number of people in the offices. And in any case, it was permutation work. If there are three of you in a department, one has to work from such and such an hour to such and such an hour, the other comes to work, so that there's no contamination of people”. (KI 7, PRONANUT, Kinshasa).

With regard to treatment, some TNUs were supplied with RUTF by the healthcare system, even though the majority (3/4) reported that they did not receive support during COVID-19 pandemic. Development and use of local RUTFs helped a lot.

…Malnourished children treated with locally produced RUTFs that we used in our TNU gain significantly more weight compared to those on Plumpy'nut. We are tempted to promote our own locally produced RUFTs (KI 53, TNU, Kinshasa).

In addition, PRONANUT organized capacity-building training courses on the nutritional management of COVID-19 and non-COVID-19 patients.

“During the COVID-19 period, for example, we were given the RUTFs I just mentioned. We were told that these were RUTFs that we financed during this COVID-19 period. If a child got sick, you’d give them this right away. That's the COVID-19 context. But also, we often received briefings regarding the care, the organization of this service during the COVID-19 period”. (KI 48, PRONANUT, Haut-Katanga).

With regard to preventive strategies (screening and nutrition education), briefings were organized with nutritionists, focusing on preventive measures against COVID-19 such as cleaning the height gauge after each use and proper use of the MUAC; and use of the double scale (for mother and child, with the possibility of cancelling the mother's weight). Nutritional education was organized for each mother and child, without bringing all the mothers together; sometimes nutritional education was organized for the mother alone. Healthcare facilities were equipped with double-weighing scales to prevent the exchange of children's underpants.

“That's how we got double-weighing scales. To prevent disease spread, only the mother holds the child, no shared panties. I cancel the mother's weight to get the child's alone”. (KI 9, PRONANUT Kinshasa).

In addition, respondents stated that PRONANUT, via the health zones, had provided numerous kits involving scales, MUAC, medicines such as amoxicillin, ampicillin, vitamin A, Plumpy'nut and therapeutic milk. In addition, a number of presentations on anthropometric measurement techniques and data collection and sharing were organized for providers.

At the local level, the focus was more on implementing instructions received from the national and provincial levels on the organization of nutritional activities. These measures should be contextualized by the TNU, taking into account their specific features. These measures included, for example, rapid registration of the mother–child pair (identity and anthropometric measurements), the use of more rooms and sites for vaccination to limit contact between patients, the use of double scales (for mother and child, with the option of cancelling out the mother's weight); the introduction of community-based care, and a limit of less than 20 mother–child pairs on the day of the growth monitoring promotion.

“We continued to vaccinate but made it compulsory to wear a mask”. (KI 50, Health zone, Kongo-central).

“Since we had these briefings on management in TNU during the COVID-19 period, since we had this information, until today, we’ve never had a case of malnutrition become a positive case of COVID-19, because we respected what we were told”. (KI 22, TNU, Haut-Katanga).

### 3.6 Theme 6: information systems

The data from the operational level goes back up each month and a three-month window remains open to allow therapeutic nutritional units to go back up with the quarterly data.

As COVID-19 started towards the end of the first quarter, the data was no longer available, resulting in missing data from April 2020 to August 2020.

“For the data to be available, the children must be screened and treated. Screening involves weighing the children and measuring their brachial perimeter with a MUAC tape. However, during COVID-19, it was forbidden to use the same panties to weigh more than one child or the same MUAC for more than one child. With these measurements, the data was therefore non-existent, so there was no data feedback”. (KI 16, PRONANUT, Kinshasa).

There were investments by donors to improve reporting and online supervisions for tracking data.

“One international non-governmental organization (NGO) provided [internet] modems and tablets for strengthening reporting of other prevailing diseases and also for use during teleconferences. Each Thursday, we continue supervision using Zoom or Teams for tracking some data and reports. But during the first peak, no specific action was done”. (KI 55, Kinshasa) and (KI 17, PRONANUT Kinshasa).

Table 2 summarizes key challenges identified and preventive strategies for maintaining NS during COVID-19 pandemic.

**Table 2:** Key challenges and preventive strategies to maintain nutrition services during COVID-19 pandemic.

Number	Pillar of the health system	Challenges identified during the pandemic	Preventive strategies
1	Leadership and governance	Nutrition was not a pillar of the COVID-19 response	Advocacy for adding nutrition services as a pillar of COVID-19 response
2	Workforce	Reduced number of nutrition services providers.	Drawing up timetables with a system of staff rotation at the facility-level. Training of community health workers for community activities without health providers’ support.
3	Financing	Lack of funds for activities to promote continuity of nutrition services (e.g., nutrition communications).	Support of some national or/and international NGOs.
4	Services delivery	Obstacles to physical access to growth monitoring promotion and community-based nutrition due to mobility restrictions	Rapid registration of the mother–child pair (identity and anthropometric measurements), the use of more rooms and sites for vaccination to limit contact between patients, the use of double scales (for mother and child, with the option of cancelling out the mother's weight); the introduction of community-based care, and a limit of less than 20 mother–child pairs on the day of the GMP
		Inability of facilities to reach the exposed target for certain antigens in the immunisation schedule.	
		Disruption of existing service delivery models	
5	Infrastructure and resources	Disruption to the global supply chain leading to supply disruptions of RUTF (Plumpy’nut)	Development and use of local foods (RUTF)
6	Information systems	Poor monitoring of data during COVID-19	Online supervisions Digitalization

Table 2 summarizes the key challenges and corresponding preventive strategies related to nutrition services across six pillars of the health system during the COVID-19 pandemic.

## 4 Discussion

The study revealed that nutrition services were significantly affected by COVID-19. Key challenges included the exclusion of nutrition as a core pillar in the COVID-19 response (being considered as a **subdivision** of the medical care committee), the reduction in the number of nutrition services providers, and insufficient funding to sustain activities promoting service continuity, such as nutrition communication campaigns. Mobility restrictions created obstacles to physical access to community-based nutrition services and to growth monitoring promotion. Health facilities also faced challenges in reaching immunization targets for certain antigens due to services delivery disruptions. Furthermore, global supply chain disruptions resulted in shortages of RUTF, such as Plumpy'nut, while poor data monitoring hindered effective decision making during the pandemic. To address these issues, several strategies were implemented. Advocacy efforts were made to include nutrition services as a key pillar in the COVID-19 response (acting as a sub-committee of the medical care committee). Timetables with staff rotation systems were introduced at the facility level to ensure continuity of services. Community health workers were trained to conduct activities independently, without direct support from healthcare providers. National and international NGOs also provided crucial support to fill gaps in service delivery. Community-based care models were introduced, and clinic visits were capped at fewer than 20 mother–child pairs per day to reduce crowding. Efforts were also made to develop and utilize locally produced therapeutic foods (local RUTF alternatives).

Nutrition is often excluded or not prioritized in emergency health responses, despite the essential role it plays in maintaining health during crises (26). During COVID-19 or Ebola for example, many countries did not include nutrition services as an essential component in their response plans (27). Responses to epidemics tend to focus primarily on controlling the spread of the disease and managing acute medical cases (28–30). This often diverts resources from essential services such as nutrition, even though it is well known that malnutrition weakens immunity and can increase vulnerability to infections (31). International organizations such as UNICEF, WHO and the World Food Programme have drawn attention to the insufficient importance attached to nutrition during crises (32), advocating for national nutrition leaders to integrate nutrition into emergency preparedness and response plans as shown in the study.

Our findings demonstrate that the number of nutrition services providers decreased significantly during the COVID-19 pandemic. Our findings are similar to what was reported in other epidemics such as Ebola (27). A study also observed that, during the COVID-19 pandemic, the reallocation of health staff to tasks specific to COVID-19 led to a reduction in the availability of staff for routine nutrition and other essential services, such as growth monitoring and treatment of malnutrition (33). We report that TNUs adopted staff rotation schedules to ensure the continuity of services while minimizing exposure to COVID-19 among health providers, as also reported elsewhere (34, 35). In other settings, tasks traditionally performed by specialized nutrition professionals were delegated to general healthcare workers or community health workers after appropriate training (36). The community relays have also been equipped to monitor growth using simplified tools such as MUAC measurement tapes (37). Teleconsultations, mobile applications and AI technologies offered accessible and cost-effective plateforms for delivering nutrition interventions, potentially reaching a broader audience compared to traditional methods. Many nutrition-related applications were complemented by tools such as activity trackers and smart scales enhancing the ability to monitor and improve lifestyles habits (38). Virtual platforms have also facilitated ongoing training for healthcare providers during movement restrictions (39). Countries have developed emergency response plans to rapidly recruit and deploy additional staff in the event of an epidemic (40). Programs have been put in place to cross-train workers in several health services, including nutrition, to increase the flexibility of staff during crises (41). Future efforts should focus on scaling up such solutions and integrating them into emergency preparedness plans to address similar challenges in the future.

During COVID-19, the lack of funding to support activities that promote continuity of services, including nutrition programmes, has been a major challenge (35). Investment in nutrition during the COVID-19 pandemic was inadequate, with funds being diverted to immediate responses to the pandemic (42). This led to disruptions in programs such as malnutrition treatment, community feeding initiatives and nutrition education campaigns (42). The World Bank and other organizations have stressed the need for sustained, multi-sector investment in nutrition during crises. They argue that, without adequate funding, the long-term health effects of malnutrition, particularly in children, could outweigh the direct effects of the epidemic itself (43). Additionally, other sectors beyond the Ministry of Health proved to be useful, such as the Ministry of Agriculture that ensured food production to prevent shortages ad price inflation (44) or supported small-scales farmers through subsidies and technical assistance (45). Education sector provided a platform for public awareness campaigns on balanced diets and food safety (46). Social protection and humanitarian aid sector partnered with international organizations to sustain nutrition programs (46). Information and communication sector supported with the use of mobiles applications and AI to provide nutrition education and dietary monitoring (38).

The lack of funding affected activities such as nutrition communication campaigns, training of health workers and the purchase of essential supplies such as RUTF (47). Untimely stock shortages of RUTF significantly damaged the case management of malnourished children. The situation was the same in several African countries (48). Local solutions were developed. One observation is that several local RUTFs were developed without laboratory support. The question to ask is regarding the composition of these different products (macro and micronutrients). The procurement of these alternative formulations will be subject to an assessment of their suitability for the management of severe wasting following the updated guidelines from WHO to support their use. The Codex Alimentarius Commission adopted the codex guidelines for RUTF on 1 November 2022 (49). The guidelines stipulate the nutritional, food safety, and contextual use of RUTF and contains several important updates to the now superseded UN Joint statement “Technical annex”. The guidelines provide a framework for new formulae made from seeds, cereals and legumes that could be sourced locally or regionally (34). Studies show that there are gaps in macronutrients. The ideal will be rapprochement during periods of calm, for example universities and these structures of case management or Ministry of Health to develop and prove the effectiveness of local products to be used as local alternatives in the event of a pandemic or crisis. Several countries produce RUTFs locally. In Indonesia, for example, new RUTF formulas have been made using local protein sources, resulting in milk-based, legume-based, fish-based, and soy-fish-based RUTFs (50–52). Among these, milk-based RUTF was preferred, therefore it was enhanced with a vitamin and mineral premix to meet WHO, UNICEF, and FAO standards (Codex Alimentarius). Additionally, a study in northeastern Uganda in January 2018 which compared a locally produced RUTF (called METU-2), made from sorghum, peanuts, honey, and ghee, with standard RUTF (Plumpy'nut), revealed that METU-2 provided a macronutrients profile similar to Plumpy'nut and could be effective in treating SAM (53, 54).

Our findings revealed that nutrition service delivery was also affected. Community services have been affected by staff absenteeism. We observed a large number of children having to benefit from either preventive or promotional services such as community-based nutrition services and to growth monitoring promotion. This situation was also found by other authors (55). Adaptative strategies in our study included rapid registration of the mother–child pair (identity and anthropometric measurements), the use of more rooms and sites for vaccination to limit contact between patients, the use of double scales (for mother and child, with the option of cancelling out the mother's weight). In other African settings, adapting measurement techniques to reduce contact with health providers were used. In Zambia, for example, Trained staff provided verbal instructions to caregivers, allowing mothers to measure their children's height, weight, and mid-upper arm circumference (MUAC) without direct contact. The study found no significant differences between caregiver and assessor measurements, suggesting this method as a viable alternative during situations requiring minimal contact (56). The introduction of community-based care, and a limit of less than 20 mother–child pairs on the day of the Growth Monitoring Program and development are strategies to be capitalized on. In Burkina Faso, nutrition programs adjusted their operations by limiting the number of participants in outpatient malnutrition centers. This strategy aimed to reduce crowding and maintain social distancing during service delivery (14).

As a matter of routine, the TNUs, community-based nutrition services and to growth monitoring promotion report their data to the central office, the central office to the province and the province to the national level (21). This reporting system was also affected during the COVID-19 period. Other studies showed that during the COVID-19 pandemic, the collection, analysis and use of nutritional data faced significant challenges, which had an impact on the ability to monitor and respond to malnutrition effectively (57). Health and nutrition surveys, including anthropometric assessments, have been delayed or cancelled due to mobility restrictions and social distancing measures. This has created gaps in real-time data on malnutrition and food security. Routine data collection systems in community nutrition programs, such as growth monitoring and screening for acute malnutrition, have been disrupted due to the reduction or cessation of many services (58). Quality of data has often been compromised due to reduced supervision, staff shortages or the use of indirect methods instead of direct anthropometric measurements (59). Some countries have adopted digital platforms and mobile phone surveys to continue collecting data on nutrition, although these methods have limitations in terms of reliability and coverage (60). Platforms such as UNICEF's NutriDash and other global dashboards were used to track nutritional data, share information and guide interventions during the pandemic (61). COVID-19 has shown the need for resilient and adaptable nutrition information systems capable of functioning during crises. Improved integration of digital technologies, capacity building for local data collection, and sustained funding for nutrition monitoring systems will be critical to address these challenges and strengthen responses in the future. Kenya, for example, expanded the use of digital platforms to monitor maternal and child nutrition indicators during the pandemic. The country utilized mobile applications and electronic health records to facilitate real-time data collection and analysis (62). Rwanda employed digital tools and crowdsourcing approaches to generate high-frequency data for diet quality monitoring at the population level. These innovations enabled timely assessments of dietary patterns and informed nutrition interventions during COVID-19 (63).

### 4.1 Strengths and limitations of the study

One of the strengths of our study is the in-depth analysis of the continuity of a specific health service using the six components of a health system. Nevertheless, excluding patient perceptions may have limited data comprehensiveness which future studies should consider.

### 4.2 Public health implication

The continuity of nutrition services in emergency situations is crucial for public health. Disruptions in these services can lead to increased morbidity and mortality, mainly in children under five.

Maintaining nutrition services means that people can continue to receive the care they need even during epidemics or pandemics.

## 5 Conclusions

COVID-19 significantly disrupted nutrition services in the Democratic Republic of Congo, impacting all six pillars of the health system. The initial exclusion of nutrition from the national COVID-19 response, insufficient funding, and restrictive public health measures contributed to the reduction of service access, workforce availability, supply chain disruptions, and data reporting challenges. However, the pandemic also served as a catalyst for adaptation and innovation at multiple levels of the health system. To strengthen preparedness for future public health emergencies, several adaptive strategies identified in this study should be institutionalized and integrated into national emergency preparedness and response plans. These include: Rapid registration systems for mother–child pairs with anthropometric measurement protocols; Expansion of service points (e.g., additional rooms for vaccinations and growth monitoring); Use of double-weighing scales to minimize contact and preserve dignity; Limiting crowd sizes to reduce exposure risk during GMP sessions; Community-based care models that empower health workers at the grassroots level and Improved integration of digital technologies.

Moreover, the pandemic highlighted the critical need for sustained domestic and donor funding dedicated specifically to nutrition services not just during crises, but as a long-term investment in health system resilience. Funding must prioritize capacity-building, logistics, and outreach services.

Equally essential is the development and scale-up of locally produced, standardized RUTFs. Local production offers a viable solution to mitigate international supply chain disruptions, but it requires laboratory validation to ensure nutritional adequacy and safety in line with Codex Alimentarius guidelines. As emergencies become more frequent and complex, integrating these lessons into nutrition and health policy will be key to ensuring continuity.

The key findings are summarized in Table 1.

## Data Availability

The original contributions presented in the study are included in the article/Supplementary Material, further inquiries can be directed to the corresponding author.

## Footnotes

^1^John Hopkins University. COVID-19 dashboard by the center for systems science and engineering [Internet]. Global map coronavirus resource center, 2020. Available: https://coronavirus.jhu.edu/map.html.

^2^ https://www.medbox.org/document/drc-consultation-prescolaire-cps-manuel-dorientation

^3^ https://medbox.org/document/drc-alimentation-du-nourrisson-et-du-jeune-enfant-fiches-techniques

^4^ https://www.medbox.org/index.php/dl/5e148832db60a2044c2d3d5c

^5^ https://www.scribd.com/document/687259181/pcima-rdcongo-pcima-17-03-2022-af

^6^ https://www.medbox.org/document/drc-consultation-prescolaire-cps-manuel-dorientation

^7^ https://www.medbox.org/document/drc-consultation-prescolaire-cps-manuel-dorientation

^8^ https://www.medbox.org/index.php/dl/5e148832db60a2044c2d3d5c

^9^ https://www.scribd.com/document/687259181/pcima-rdcongo-pcima-17-03-2022-af

^10^ https://www.scribd.com/document/687259181/pcima-rdcongo-pcima-17-03-2022-af
