# Supplementary material for: Challenges and strategies for maintaining nutrition services in the Democratic Republic of Congo during COVID-19: a qualitative study
Source: Front Health Serv. 2025 Jun 24;5:1551131. doi: 10.3389/frhs.2025.1551131 (PMC12234544; doi:10.3389/frhs.2025.1551131)
Supplement: Supplementary file 1 [file Table1.docx]

**GUIDE D’ENTRETIEN POUR LES SERVICES DE NUTRITION**

**THEME 1 : ACTIVITES DE NUTRITION**

1. **Identifiants**

a) Sexe :

b) Institution :

c) Durée du poste :

d) Profession :

e) Bureau / poste :

f) Téléphone :

g) Quel a été votre rôle dans la réponse au COVID-19 ? Si applicable

h) Votre fosa a-t-elle une UNTA ? UNTI ? Les deux ?..........................

2. En réponse à la pandémie de COVID-19 dans le pays, plusieurs Interventions Non Pharmaceutiques (INP) ont été mis en œuvre sur la base desquels je voudrais vous poser quelques questions. Premièrement, lesquels de ces INP ont été mis en œuvre dans le pays ?

a) Confinement

b) Restrictions de mouvement (couvre-feu, transports publics et privés)

c) Quarantaine (institutionnelle et individuel) et isolement

d) Fermeture d'écoles ou d'établissements d'enseignement

e) Fermeture temporaire ou réglementée des lieux de rassemblements sociaux (lieux de culte, marchés, bars et événements sociaux tels que mariages, enterrements).

f) Distanciation sociale

g) Individuel ou personnel (lavage fréquent des mains avec du savon ou utilisation de désinfectant pour les mains, port obligatoire d'un masque facial, étiquette respiratoire)

h) Restrictions de voyage (telles que les restrictions de vol et les mouvements aux frontières)

i) Interdire les rassemblements de masse (tels que les mariages et les funérailles)

j) Recherche des contacts

k) Modifications des politiques relatives aux prisons

Demandez à l'informateur ce qui suit :

1. Comment se déroule la Consultation préscolaire (CPS) temps normal (période avant Covid-19) ? Comment se déroule la Consultation préscolaire (CPS) en temps de Covid-19 ?

Quels sont les effets positifs de l’implémentation de chacune des interventions non pharmaceutiques citées ci-haut sur la CPS ?

Quels sont les effets négatifs de l’implémentation de chacune de ces interventions non pharmaceutiques sur la CPS ?

1. Comment se déroule les activités de NAC (Nutrition a assise communautaire) temps normal (période avant Covid-19) ? Comment se déroule les activités de NAC en temps de Covid-19 ?

Quels sont les effets positifs de l’implémentation de chacune des interventions non pharmaceutiques citées ci-haut sur les activités de NAC ?

Quels sont les effets négatifs de l’implémentation de chacune des interventions non pharmaceutiques citées ci-haut sur les activités de NAC ?

1. Comment se déroule les activités de PCIMA (Prise en charge intégrée de la malnutrition aigüe) temps normal (période avant Covid-19) ? Comment se déroule les activités de PCIMA en temps de Covid-19 ?

Quels sont les effets positifs de l’implémentation de chacune des interventions non pharmaceutiques citées ci-haut sur les activités de PCIMA ?

Quels sont les effets négatifs de l’implémentation de chacune des interventions non pharmaceutiques citées ci-haut sur les activités de PCIMA ?

1. Qu’est-ce que le niveau national, provincial et opérationnel ont fait pour minimiser les effets négatifs de ces interventions non pharmaceutiques et assurer de ce fait la continuité du fonctionnement des services de nutrition ?
2. Quelles sont les structures locales qui ont travaillé pour maintenir les services ? Y-a-t-il eu des campagnes de masse ou autres choses pour encourager les parents d’enfants malnutris à utiliser les services ?
3. Comment les personnels des services de nutrition ont-ils été réaffecté ? le nombre actuel des personnels est-il suffisant ?
4. Quelles sont les stratégies qui ont marché ? lesquelles n’ont pas marché ? lesquelles sont innovantes ?
5. Que pensez-vous de l’appui financier des services de nutrition avant Covid-19 et pendant Covid-19 ?
6. Recevez-vous des ressources additionnelles pour maintenir le fonctionnement de votre service ?
7. Y-a-il des problèmes dans le rapportage des données (Avant Covid-19 et pendant Covid-19) ?
8. Quelles sont les défis auxquels vous faites face dans ce service de Nutrition ?
9. Que recommanderiez-vous concernant la mise en œuvre des interventions non pharmaceutiques dans le cadre de la lutte contre le COVID-19 et les pandémies ?

**THEME 2 : MECANISMES DE RESILIENCE**

1. En essayant de vous adapter face aux ruptures de stock en Plumpynut, lait F75 ou F100,

**Aux enfants avec MAM (Malnutrition aigue modérée)**

Quels traitements donnez-vous ?

Quelle en est la composition ?

A quelle quantité ?

Pour combien de temps ?

Comment préparez-vous ces produits ?

Quelles difficultés rencontrez-vous depuis le début de la Covid-19 pour prendre en charge avec MAM ?

**Aux enfants avec MAS (Malnutrition aiguë sévère)**

Quels traitements donnez-vous ?

Quelle en est la composition ?

A quelle quantité ?

Pour combien de temps ?

Comment préparez-vous ces produits ?

Quelles difficultés rencontrez-vous depuis le début de la Covid-19 pour prendre en charge avec MAS sans complication ? MAS avec complication ?

Avez-vous un protocole autre que le protocole PCIMA ? Si oui, pouvez-vous nous en parler ? **(Demander un exemplaire)**

1. Puis-je observer le registre dans lequel vous enregistrez les paramètres des enfants de moins de 5 ans suivis chez vous par traitement local ?

Svp, choisissez au hasard 3 enfants, recopiez l’indicateur (Evolution du poids depuis l’admission jusqu’à la décharge) pour 3 enfants de moins de 5 ans pris en charge avec un produit local.
